# Supplementary figures and images for: Integration Linkage Mapping and Comparative Transcriptome Analysis to Dissect the Genetic Basis of Rice Salt Tolerance Associated with the Germination Stage
Source: Int J Mol Sci. 2024 Sep 26;25(19):10376. doi: 10.3390/ijms251910376 (PMC11476921; doi:10.3390/ijms251910376)

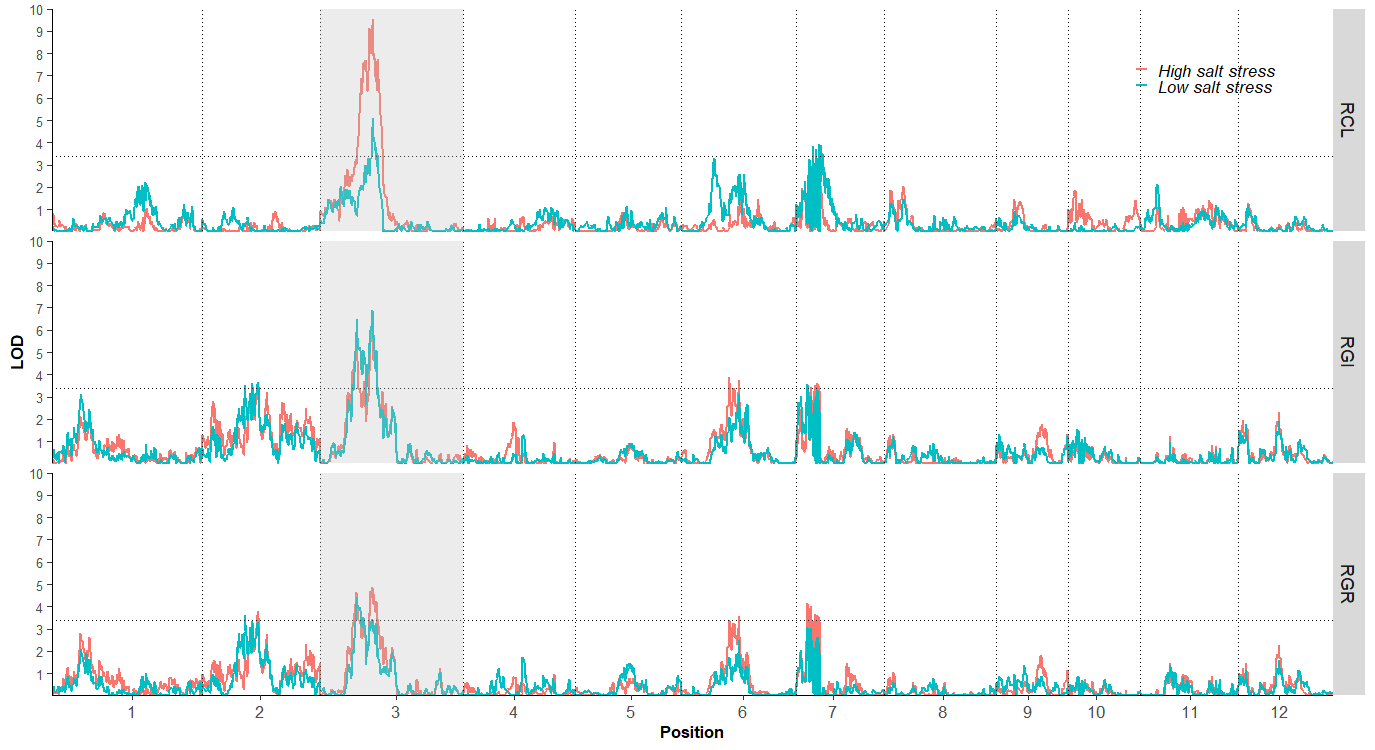

Supplement: Supplementary file 1 [file ijms-25-10376-s001.zip › Figure S2.tiff]

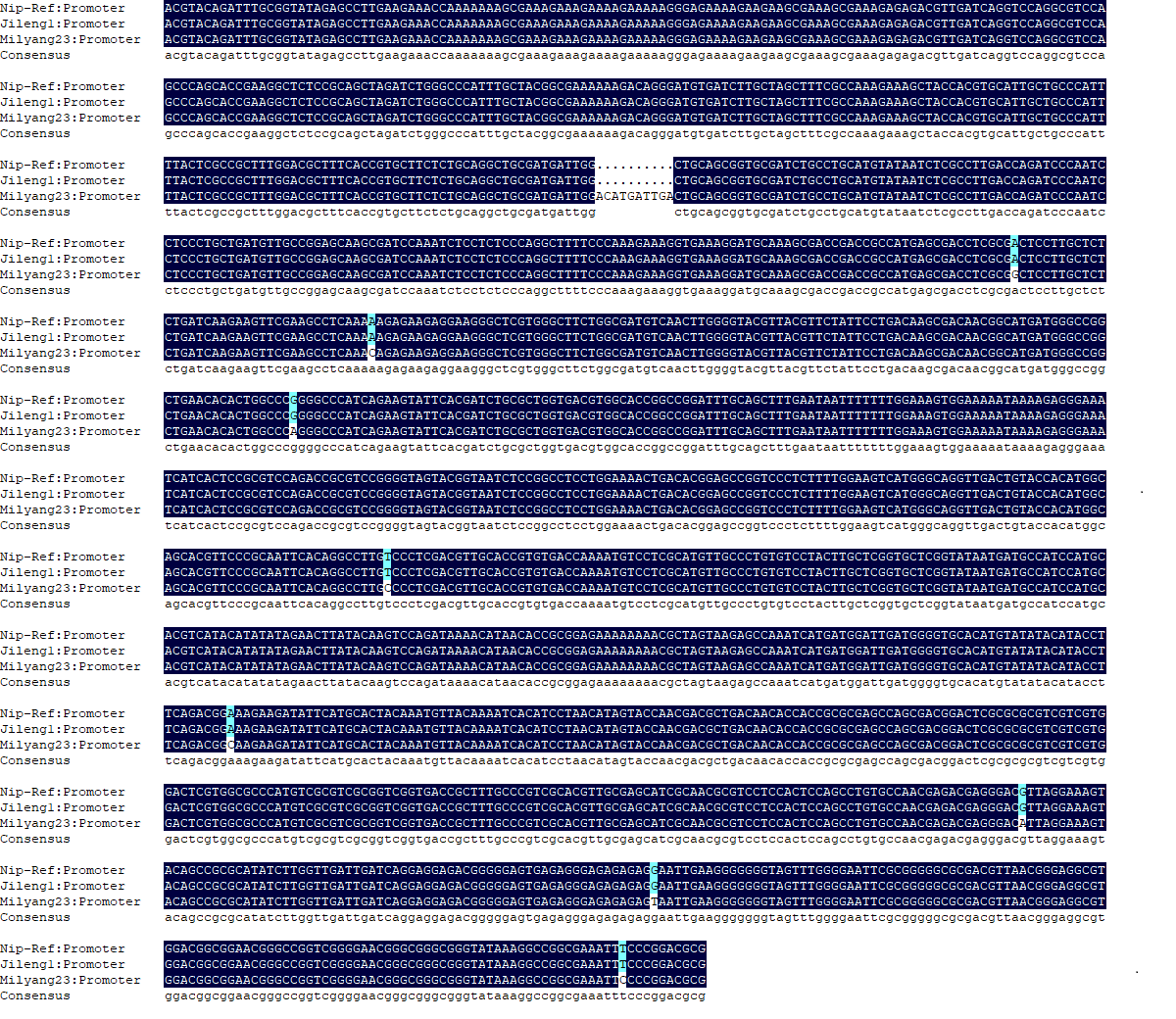

Supplement: Supplementary file 1 [file ijms-25-10376-s001.zip › Figure S3.tif]

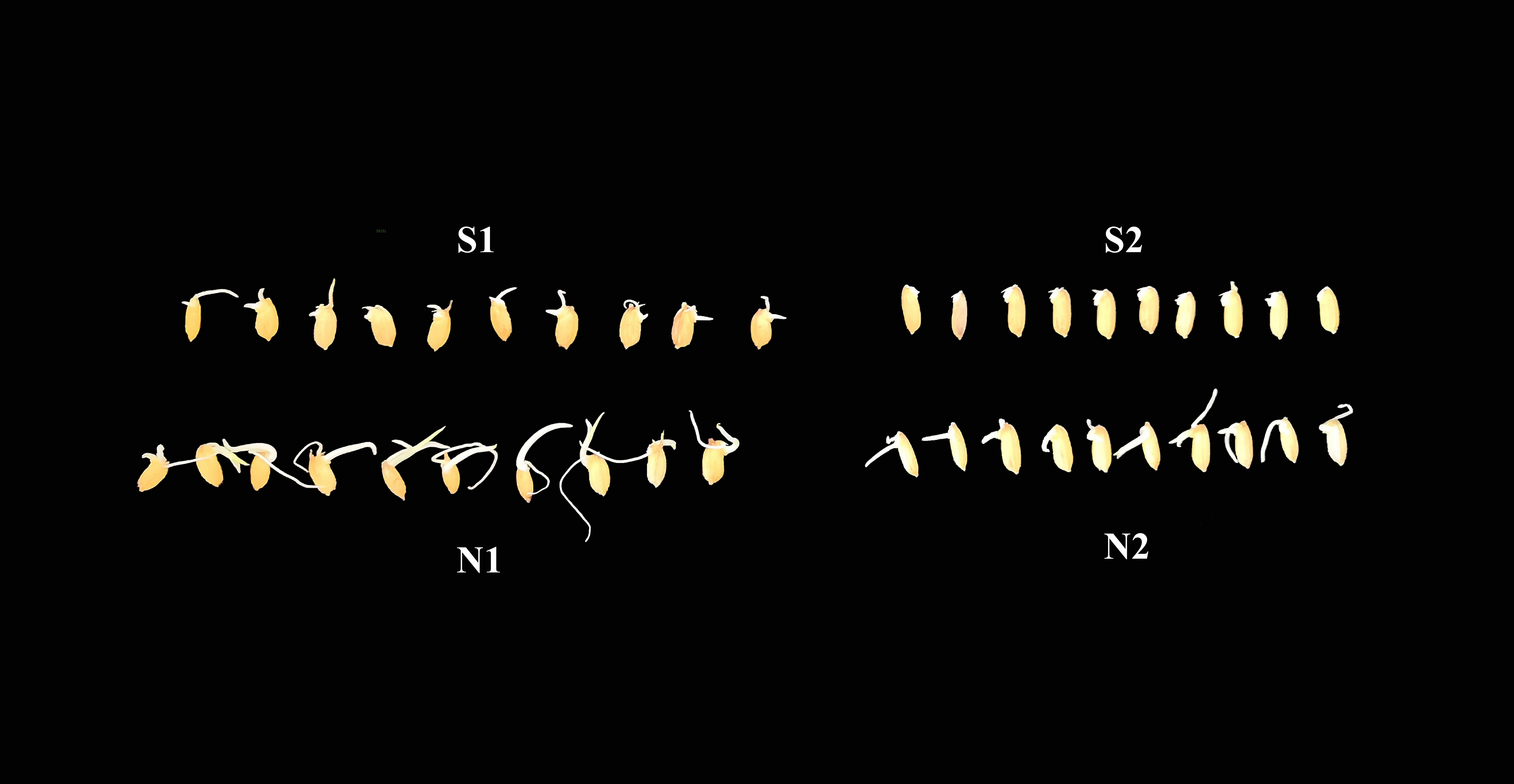

Supplement: Supplementary file 1 [file ijms-25-10376-s001.zip › Figure S1.tif]
